# Regional Differences and Mortality-associated Risk Factors among Older Patients with Septic Shock: Administrative Data Analysis with Multilevel Logistic Regression Modelling

**DOI:** 10.31662/jmaj.2024-0331

**Published:** 2025-06-06

**Authors:** Shinichiro Yoshida, Akira Babazono, Ning Liu, Reiko Yamao, Reiko Ishihara

**Affiliations:** 1 Graduate School of Medical Sciences, Kyushu University, Fukuoka, Japan; 2 Department of Health Care Administration and Management, Faculty of Medical Sciences, Kyushu University, Fukuoka, Japan; 3 Department of Preventive Medicine and Community Health, University of Occupational and Environmental Health, Kitakyushu, Japan; 4 Department of Human Sciences, Faculty of Human Sciences, Osaka University of Economics, Osaka, Japan

**Keywords:** intensive care unit, septic shock, older adults, mortality, regional variability, context effect, board-certified intensivist

## Abstract

**Introduction:**

Variations in intensive care unit (ICU) policies and physician characteristics influence mortality, potentially leading to regional differences in mortality rates. Previous studies have not specifically focused on septic shock or older patients and have lacked consideration of the context effect. We hypothesized that regional variability in mortality exists among older patients with septic shock and investigated factors associated with mortality.

**Methods:**

Administrative medical claims data were analyzed. Participants were enrolled from April 2015 to March 2020 in Fukuoka Prefecture, Japan. ICU physicians were classified based on board certification in intensive care medicine as either “intensivists” or “ICU-dedicated physicians”. The primary outcome was 28-day mortality after ICU admission. Data from all ICUs in Fukuoka Prefecture and 9 secondary medical areas were analyzed. We calculated and compared the 28-day mortality rates across regions. Multilevel logistic regression analyses were conducted to adjust for the context effect.

**Results:**

Among the 1,238 participants, mortality across regions ranged from 18.3% to 41.4%. Based on multilevel logistic analyses, age, sex, postsurgical admission, and the number of ICU beds per intensivist were significantly associated with mortality. The adjusted odds ratio from the multilevel analysis for having no intensivists versus having ≥1 intensivist per 4 ICU beds was 1.99 (95% confidence interval 1.15-3.44, p = 0.01).

**Conclusions:**

After accounting for the regional context effect, our analysis confirmed regional mortality variability in mortality among older patients with septic shock. Mortality was influenced by whether ICU physicians are board-certified in intensive care medicine. These findings suggest that sufficient commitment in terms of time, intensity, and knowledge is crucial to reducing mortality in older patients with septic shock.

## Introduction

Treatment of septic shock requires clinical experience, knowledge, and multidisciplinary care, and its outcome reflects the quality of the intensive care provided ^(1)^. Despite a decrease in recent years, the incidence of septic shock-related mortality remains high ^(2), (3), (4)^, especially in older patients ^(5), (6)^. Various factors influence intensive care unit (ICU) mortality; for example, closed ICU management, high-intensity intensivist consultation ^(7), (8), (9), (10), (11)^, physician staffing ^(7), (12), (13)^, ICU admission rate ^(14)^, racial and ethnic minority status ^(15), (16), (17)^, and insurance type ^(18)^ have all been associated with ICU mortality. Given that these factors are difficult to optimize across hospitals, ICU mortality rates may vary between hospitals and regions. However, previous studies have not examined septic shock-related mortality by region while accounting for potential regional contextual effects, which could introduce bias. Determining whether variations in mortality statistics by region are attributable to the contextual effects of the region or the hospital can provide evidence for the need for region-specific efforts or comprehensive improvement in ICU operations.

Most patients with sepsis are older; older patients are vulnerable and have poor outcomes, which may reflect the inadequacy of treatment. Therefore, we believe that older patients with septic shock have greater opportunities for improved ICU treatment and are particularly suitable targets for assessing the quality of ICU care.

This study examined whether the variations in regional mortality statistics for septic shock in older patients were due to regional or hospital context effects. After accounting for these effects, we conducted a comprehensive examination of the impact of multiple variables on mortality. The independent variables included several ICU requirements in Japan, such as ICU resource availability and staffing levels.

## Materials and Methods

### Study design and participants

We conducted a retrospective open-cohort study using administrative claims data of older patients who were beneficiaries of the Fukuoka Prefecture Wide-Area Association of Latter-Stage Elderly Healthcare in Japan. In western Japan, Fukuoka Prefecture has a population of approximately 5,101,000 people. In 2018, this health insurance program covered approximately 606,000 individuals in Fukuoka Prefecture, accounting for about 95% of residents aged 75 years or older (Supplementary Table 1). Under Japanese health policy, all individuals aged 75 years or older are insured.

Administrative claims data contain various types of information, including the dates and details of medical treatments, such as surgical procedures and prescribed medications. Furthermore, the dataset encompasses personal information, including date of birth and sex, as well as the rationale behind the acquisition and loss of health insurance qualifications. In addition, the dataset includes the names of diseases affecting patients, along with the year and month of diagnosis; however, it does not include exact diagnosis dates.

**Figure 1.**
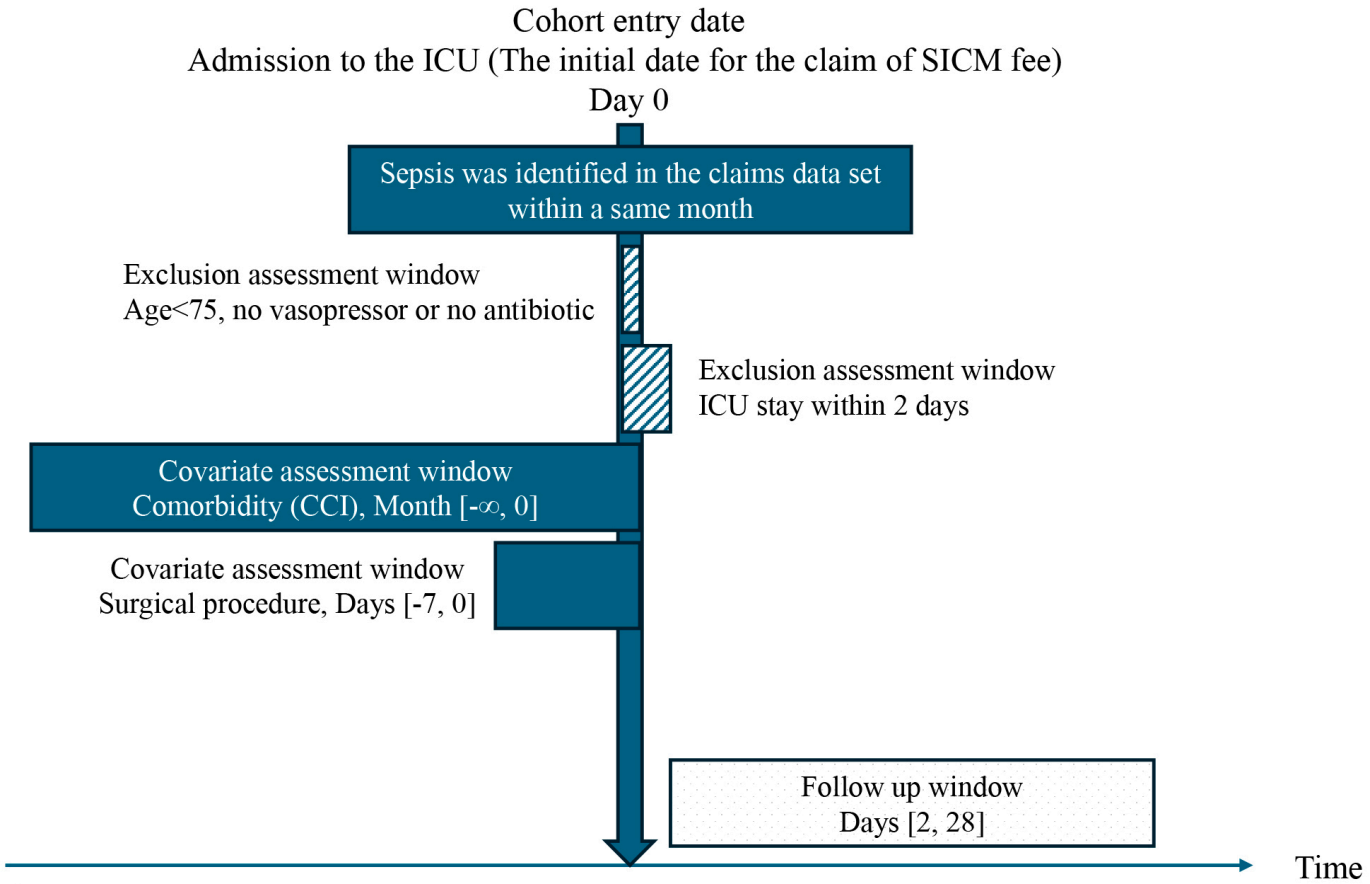
Graphical description of the study cohort The diagnosis was identified solely by year and month, as recorded in the administrative claims data. Consequently, patients with sepsis were included if the diagnosis was registered at the time of ICU admission. Comorbidities were extracted if they were registered within the same month as or before the sepsis diagnosis. The inclusion criteria for age, vasopressor use, and antibiotic use were met by the date of ICU admission. The term “ICU admission for sepsis with surgery” was defined as any surgical procedure performed on or within 7 days before ICU admission. ICU: intensive care unit.

We screened patients aged ≥75 years who were admitted between April 2015 and March 2020 to all adult ICUs in Fukuoka Prefecture. ICU admission was identified based on the reimbursement of a specialized intensive care management (SICM) fee (here, “specialized” does not refer to specific diseases such as stroke and acute coronary syndrome). We identified patients who were admitted to the ICU for more than 1 day. Thereafter, we selected eligible patients using codes specific to health insurance claims in Japan, including the terms “sepsis” or “septic” (Supplementary Table 2). Among the patients who met these criteria, we applied a modified method from previous studies ^(19)^ and included only those who received any vasopressor or antibiotic treatment during ICU admission. Studies that have used administrative data on sepsis included diagnostic names, antibiotic use, and blood culture results. Since blood cultures are not always performed in practice, our inclusion criterion was the presence or absence of vasopressor administration, with a particular focus on septic shock treatment. We excluded data from patients who lost their health insurance qualifications for reasons other than death (e.g., relocation outside Fukuoka Prefecture) because their outcomes could not be assessed. Records of second or later ICU admissions, multiple admissions for septic shock, and hospitalizations outside Fukuoka Prefecture were also excluded (Figure 1).

Although we did not investigate ICU format and intensity, we used the Japanese Society of Intensive Care Medicine (JSICM) board-certified physician list ^(20)^ to accurately determine the number of JSICM board-certified physicians at each hospital.

In this study, “intensivist” refers to a physician with JSICM board certification in intensive care, whereas “ICU-dedicated physician” refers to an ICU physician without a JSICM certification in intensive care. The term “ICU physician” encompasses both.

The number of hospital beds in this study ranged from 150 to 1,275, as described in a report on the medical functions of hospital beds in Fukuoka Prefecture ^(21)^.

This study was approved by the Institutional Review Board of Kyushu University (Clinical Bioethics Committee of the Graduate School of Medical Sciences, Kyushu University [approved number 2021-335]), which waived the requirement for informed consent for this non-interventional study because only anonymized dataset information was analyzed.

### ICU settings in Japan

SICM fee claims can be submitted by hospitals if their ICU meets government-stipulated conditions. The 2 types of SICM fees―ordinary and superior (or “standard ICU” and “resource-rich ICU”, respectively ^(22)^)―require full-time availability of in-house dedicated physicians in the ICU and an ICU bed/nurse ratio ≤2. Additionally, the number of cases and devices account for approximately 70% of all ICU cases. A superior SICM fee requires 2 or more dedicated ICU physicians with at least 5 years of experience in intensive care medicine, a fully available in-house clinical engineer, and an experienced intensive care nurse for at least 20 hours per week. An ordinary SICM fee does not require professionals specialized in intensive care. ICU physician specialties such as anesthesiology, emergency medicine, cardiovascular medicine/surgery, neurology/neurosurgery, and abdominal surgery are not required in Japan to claim the SICM fee. In our study, we refer only to ICUs that can claim SICM fees.

### Regional and hospital profiles

The Japanese government defines a secondary medical area (SMA) as a designated region where residents can receive comprehensive conventional inpatient care based on geographical area, population, and hospital distribution. Fukuoka Prefecture has 13 SMAs, 4 of which do not have any hospitals eligible to claim SICM fees. There are 4 academic hospitals in 3 SMAs and 10 tertiary emergency medical centers in 5 SMAs that have an ICU eligible for SICM fee reimbursement.

### Primary outcome and study variables

The primary outcome was 28-day mortality after ICU admission. To quantify variations in mortality attributable to patient characteristics or hospital profiles, we conducted a multilevel logistic analysis after performing univariate and multivariate analyses. Patient characteristics included age, sex, year of admission, and the Charlson Comorbidity Index (CCI). The CCI was calculated as the sum of scores for the presence or absence of major organ dysfunction and diseases. Major diseases included in the CCI were cardiac, cerebrovascular, chronic pulmonary, and collagen diseases; diabetes; liver dysfunction; and malignancy. The CCI is widely used in studies involving administrative claims data because it correlates with mortality risk and allows for relative ease of data extraction and disease scoring. In this study, a score of 0-1 was classified as mild disease, 2-6 as moderate disease, and ≥7 as severe disease. Patients who underwent surgical procedures within 7 days prior to ICU admission were classified as postoperative admission cases. Hospital-related factors included location, number of hospital beds, proportion of ICU beds within each hospital, and the type of SICM fee claimed. Additionally, ICU bed-to-intensivist ratio (intensivist density) was examined, although the exact number of ICU beds per ICU physician could not be determined. We stratified the intensivist density into 3 groups, ensuring that each contained approximately an equal number of ICUs. To compare regional frequencies, we also investigated other variables, including the use of broad-spectrum antibiotics (carbapenems), antibiotics for methicillin-resistant *Staphylococcus aureus*, and vasopressors, as the usage and rates of these agents may vary among ICU physicians.

### Statistical analysis

We compared participants’ characteristics across SMAs. For dichotomous variables, the chi-square (χ^2^) test was used to assess regional differences, whereas for continuous variables, the Kruskal-Wallis test was performed. Mortality was compared using odds ratios derived from univariate logistic regression analysis, with SMA as the explanatory variable. Although each variable was analyzed under the assumption of independence, we tested for multicollinearity by calculating the variance inflation factor (VIF) to ensure the validity of this assumption.

We were concerned that more severely ill patients might be treated at specific hospitals, such as ICUs in university hospitals. Moreover, we considered regional biases, such as population size, age distribution, and ICU physicians’ policies. Therefore, we examined the variance at the hospital or regional levels using a multilevel logistic regression analysis.

The null model included no variables, while Model 1 included age group, sex, fiscal year of admission, CCI, and postoperative admission as participant characteristics. In Model 2, hospital size (based on the number of hospital beds), the proportion of ICU beds to total hospital beds, intensivist density, and the type of SICM-free hospital profile were added to the Model 1 variables.

The median odds ratio (MOR) was calculated to quantify the variation or heterogeneity in outcomes between clusters using between-cluster variance. A higher MOR indicates a greater contextual effect of the model, in which case multilevel analysis is effective. Conversely, a MOR close to 1 implies very small inter-cluster variability in outcomes, suggesting that the results from the multilevel analysis would be similar to those from the multivariable logistic regression analysis.

All analyses were performed using Stata/IC 14 software (StataCorp, College Station, TX, USA). All reported p-values were two-tailed, and the level of significance was set at p < 0.05.

## Results

**Figure 2.**
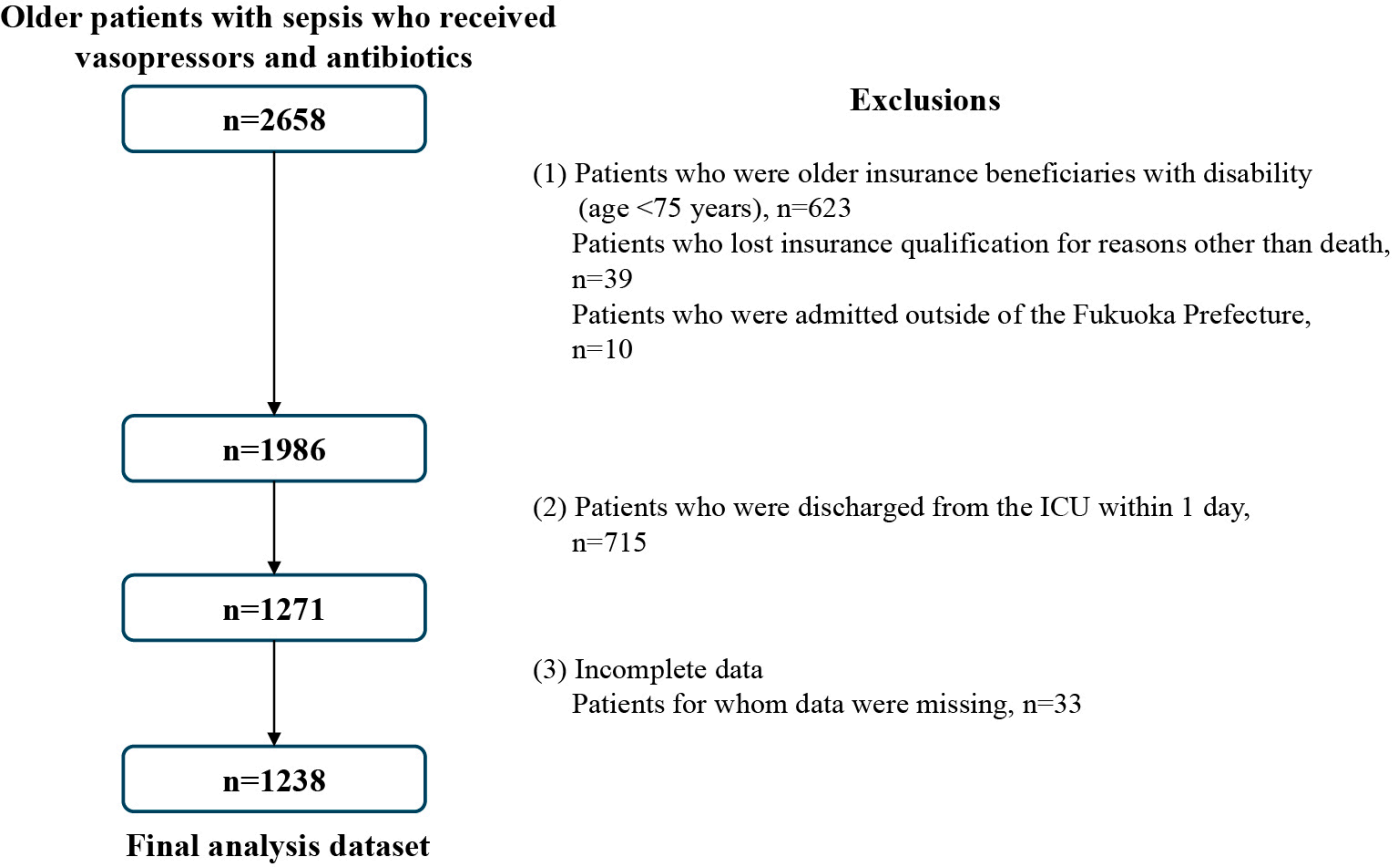
Flowchart of patient identification and selection Patients were excluded if (1) they were insurance beneficiaries with a disability (age ≥65 to <75 years), which could have prevented a fair evaluation, or had lost insurance qualification for reasons other than death, such as relocation, thereby preventing follow-up; (2) their ICU stay was too short for the quality of care to be adequately assessed; and (3) they had missing data. ICU: intensive care unit.

We identified 2,658 patients diagnosed with sepsis who received vasopressors and antibiotics. However, after excluding patients due to insurance qualifications, hospital location, short ICU stays, and missing data, the final analyses included data from 1,238 patients (Figure 2). Table 1 illustrates the distribution of ICU numbers according to the specific characteristics of each hospital and ICU.

**Table 1.** Descriptive Data of the Characteristics and Distribution of the Target Hospitals and ICUS.

Variable		Number of ICUs	(%)
Number of hospital beds	≥400	18	51.4
	<400	17	48.6
Proportion of ICU beds to hospital beds	<1.5%	12	34.3
	1.5-3%	12	34.3
	≥3%	11	31.4
ICU bed-to-board certified physician ratios	≤4	7	20
	>4	9	25.7
	No certified physician	19	54.3
Type of SICM fee	Resource rich	6	17.1
	Standard	29	82.9

ICU, intensive care unit; SICM, specialised intensive care management

Regional differences in postoperative admission, medication use, and 28-day mortality rates after ICU admission were observed (Table 2 and 3). The 28-day mortality rate after ICU admission ranged from 18.3 to 41.4% across SMAs (Table 3). The threshold for stratifying intensivist density into 3 groups was 4 ICU beds. We categorized the patients into 3 groups: no intensivist, 4 or more ICU beds per intensivist, and >4 ICU beds per intensivist. A VIF assessment indicated that all variables retained their independence, and no multicollinearity was identified (Table 4).

**Table 2.** Comparison of the Participants’ Characteristics and Treatments.

Variable	Secondary Medical Area									
	1	2	3	4	5	6	7	8	9	*p*
N	295	29	41	70	186	20	157	372	68
Age, y (median, IQR)	83 (9)	84 (10)	84 (6)	83.5 (8)	83 (8)	86 (7)	84 (8)	83 (8)	83 (7)	0.20
Sex, male (%)	57.3	65.5	53.7	48.6	60.8	50	53.5	55.1	48.5	0.55
CCI, median (IQR)	2 (2)	2 (3)	1 (2)	2 (2)	2 (2)	0 (2.5)	2 (2)	2 (2)	2 (2)	0.88
Postoperative (%)	30.5	44.8	31.7	45.7	41.4	35	34.4	42.2	25	0.01
Vasopressor use (%)									
Noradrenaline	84.4	82.8	70.7	82.9	82.8	65	91.7	86.8	83.9	0.02
Vasopressin	5.4	3.5	2.4	1.4	22	0	49.7	8.1	17.7	<0.01
Dopamine	54.6	55.2	63.4	31.4	31.2	55	21.7	38.2	36.8	<0.01
Adrenaline	6.8	6.9	4.9	7.1	9.1	5	15.9	11.6	8.8	0.09
Antibiotic use (%)									
Carbapenems	64.4	65.5	58.5	58.6	54.3	25	75.2	53.2	48.5	<0.01
Anti-MRSA agents*	9.2	10.3	2.4	4.3	18.8	5	32.5	12.6	8.8	<0.01

IQR, interquartile range; CCI, Charlson Comorbidity Index; MRSA, methicillin-resistant *Staphylococcus aureus* ^*^Anti-MRSA agents include vancomycin, teicoplanin, daptomycin, and linezolid

**Table 3.** Mortality Rates in the Secondary Medical Areas.

Secondary medical area	n	28-Day mortality (%)	OR (95% CI)	SE	*p*
1	295	32.2	2.12 (1.36-3.31)	0.482	<0.01
2	29	41.4	3.16 (1.38-7.22)	1.332	<0.01
3	41	31.7	2.08 (0.98-4.42)	0.800	0.06
4	70	25.7	1.55 (0.81-2.97)	0.515	0.19
5	186	18.3		Reference	
6	20	40.0	2.98 (1.13-7.85)	1.473	0.03
7	157	22.9	1.33 (0.79-2.25)	0.357	0.29
8	372	33.6	2.26 (1.47-3.48)	0.496	<0.01
9	68	38.2	2.77 (1.50-5.12)	0.868	<0.01

OR, odds ratio; SE, standard error; CI, confidence interval

**Table 4.** The Variance Inflation Factor (VIF) for Each Variable.

Variable		VIF	1/VIF
Age group			
	80-84	1.53	0.65
	85-89	1.51	0.66
	≥90	1.40	0.71
	≥3%	11	31.4
Sex		1.06	0.94
CCI	Moderate	1.04	0.97
	No certified physician	19	54.3
	Severe	1.03	0.97
Fiscal year	2016	1.64	0.61
	2017	1.62	0.62
	2018	1.68	0.60
	2019	1.63	0.61
Number of hospital beds		1.80	0.56
ICU bed-to-board certified physician ratios	> 4	3.80	0.26
	No certified physician	3.75	0.27
Proportion of ICU beds to hospital beds	1.5-3%	1.85	0.54
Number of hospital beds	≥3%	1.79	0.56
Type of SICM fee		1.75	0.57
Procedure		1.04	0.96
Mean VIF		1.76

CCI, Charlson Comorbidity Index; ICU, intensive care unit; SICM, specialised intensive care management

A MOR of approximately 1.0 implied homogeneity among these variables and indicated that the multilevel analysis results, which accounted for multiple hospital and medical region levels, were not significantly biased at any level (Table 5). Consequently, the general multivariable logistic regression analysis results, conducted without distinguishing between hospitals and medical regions, did not differ significantly. The full-variable model (Model 2) showed the best fit based on the log-likelihood results. The variance and intra-class correlation coefficients of Model 2 were the lowest among the 3 models. A greater decline in variance and intra-class correlation coefficients was observed in Models 1 and 2, suggesting that the additional variables included in Model 2 had a greater effect on mortality variability than those included in Model 1.

**Table 5.** Results of the Multilevel Logistic Regression Analyses.

Variable		Null Model		Model 1		Model 2	
		OR (95% CI)	*p*	AOR (95% CI)	*p*	AOR (95% CI)	*p*
Age group, y	75-79			Reference		Reference	
	80-84			1.29 (0.92-1.81)	0.14	1.28 (0.91-1.80)	0.15
	85-89			1.47 (1.04-2.08)	0.03*	1.49 (1.05-2.11)	0.03
	≥90			1.82 (1.19-2.76)	0.01*	1.86 (1.22-2.83)	<0.01
Sex	Male			Reference		Reference	
	Female			0.74 (0.57-0.96)	0.03*	0.73 (0.56-0.94)	0.02
Fiscal year	2015			Reference		Reference	
	2016			1.14 (0.76-1.70)	0.52	1.12 (0.75-1.67)	0.58
	2017			1.17 (0.79-1.75)	0.44	1.16 (0.78-1.74)	0.46
	2018			0.89 (0.60-1.32)	0.57	0.87 (0.59-1.30)	0.51
	2019			1.09 (0.73-1.63)	0.69	1.10 (0.73-1.64)	0.66
CCI	Mild			Reference		Reference	
	Moderate			1.07 (0.83-1.38)	0.60	1.10 (0.85-1.41)	0.48
	Severe			1.21 (0.35-4.17)	0.76	1.28 (0.37-4.38)	0.70
Procedure	Postoperative			0.62 (0.47-0.81)	<0.01*	0.62 (0.47-0.81)	<0.01
Number of hospital beds	≥400					Reference	
	<400					0.87 (0.61-1.25)	0.46
Proportion of ICU beds to hospital beds	<1.5%					Reference	
	1.5-3%					1.14 (0.77-1.70)	0.51
	≥3%					0.88 (0.60-1.30)	0.52
ICU bed-to-board certified physician ratios	≤4					Reference	
	>4					1.33 (0.77-2.31)	0.30
	No certified physician					1.99 (1.15-3.44)	0.01
Type of SICM fee	Resource rich					Reference	
	Standard					0.85 (0.57-1.28)	0.44
Log likelihood		−747.96		−734.35		−729.94	
Variance	SMA	0.06 (0.01-0.44)		0.05 (0.01-0.51)		0.03 (0.00-0.50)	
	Hospital	0.03 (0.00-0.43)		0.04 (0.00-0.42)		0.01 (0.00-17.07)	
ICC	SMA	0.02 (0.00-0.12)		0.02 (0.00-0.13)		0.01 (0.00-0.13)	
	Hospital	0.03 (0.01-0.09)		0.03 (0.01-0.09)		0.01 (0.00-0.07)	
MOR	SMA	1.26 (1.09-1.88)		1.24 (1.07-1.97)		1.16 (1.04-1.97)
	Hospital	1.19 (1.05-1.87)		1.22 (1.07-1.86)		1.10 (1.00-51.44)	

OR, odds ratio; CI, confidence interval; AOR, adjusted odds ratio; CCI, Charlson Comorbidity Index; ICU, intensive care unit; SICM, specialised intensive care management; SMA, secondary medical area; ICC, intra-class correlation coefficient; MOR, median odds ratio

In Model 2, the 28-day mortality after ICU admission was significantly influenced by age, sex, postoperative admission, and intensivist density (Table 5). The variables used in the multivariable logistic regression analysis were the same as those used in the multilevel logistic regression analysis. Age, sex, postoperative admission, and intensivist density were significantly associated with mortality (Supplementary Table 3).

## Discussion

We conducted a multilevel analysis to examine the possibility of contextual effects at the regional and hospital levels. The multilevel analysis results showed that the contextual effect in an SMA or hospital was small; therefore, the impact of variations in patient characteristics among our study clusters (i.e., SMA and hospitals) was minimal to determining mortality variation. However, the intra-class correlation coefficients in Model 2 decreased to less than half of those in the null model when controlling for hospital profiles. Therefore, hospital variables can affect the 28-day mortality rate after ICU admission and should be considered in future analyses. We confirmed our hypothesis that regional mortality among older patients with septic shock varied across regions and was potentially attributable to mortality-associated factors, including old age, sex, being a nonsurgical patient, and the ICU bed-to-intensivist ratio. The intensivist density of ICU beds significantly influenced mortality.

Previous studies have identified several factors contributing to variations in ICU patient prognoses. These findings could be extended to older patients with septic shock after adjusting for context effects at the regional level. The present study analyzed Japan’s unique ICU physician staffing requirements, which include 2 types of ICU physicians: JSICM-board-certified intensivists and ICU-dedicated physicians. Differences among ICU physicians in Japan include periodic certification renewal, service intensity, and professional focus. Intensivists in Japan must satisfy revalidation requirements every 5 years by conducting research, participating in learning sessions, and instructing trainees in intensive care medicine. In contrast, ICU-dedicated physicians are not required to maintain SICM accreditation. Another concern regarding ICU-dedicated physicians is the intensity of their commitment to ICU patients. ICU physicians are permitted to practice their primary specialty in addition to providing intensive care, as long as they remain in the ICU ward. Consequently, ICU-dedicated physicians may limit their involvement with ICU patients due to obligations in their primary specialty, particularly in an open ICU format, where attending physicians primarily manage ICU patients. In such situations, ICU-dedicated physicians may be less involved in direct patient care. Intensivists, whose primary specialty is intensive care, may maintain a higher level of commitment to ICU patients. Therefore, the strength of intensivists in the present study may stem from their continuous engagement in intensive care and their active commitment to ICU patients. However, our results suggest potential weaknesses in the definition of ICU physicians for the reimbursement of intensive care management in Japan.

We propose that consideration should be given to whether study outcomes, including those of previous studies, focus on ICU mortality or septic shock mortality. Recent studies ^(13), (23), (24)^ have shown that a closed ICU format and nighttime staffing of intensivists were not necessarily beneficial for patients’ outcomes after ICU admission. However, particularly in septic shock treatment, a higher patient-to-intensivist ratio and the lack of intensivists available during the night may be associated with poor outcomes ^(13), (25)^. One study ^(26)^ indicated that case volume may have a positive effect on septic shock outcomes. In view of our findings, the intensity of commitment by intensivists is likely to remain important in the treatment of older patients with septic shock. Moreover, the number of intensivists should be increased based on the number of patients. This finding is consistent with the results of a previous study ^(27)^ that showed the number of intensivists per bed was associated with ICU efficiency. Another study ^(28)^ on patient-to-intensivist ratios reported that the positive effect on mortality was optimized at 7.5. This value was obtained from a case-mixed population and may have been influenced by the number of board-certified intensivists treating older patients with septic shock. Based on our findings, the intensity of ICU physicians’ commitment to older patients with septic shock remains an important factor. Moreover, we stratified the cohort using a 4-bed intensivist ratio to enable a comparable number of hospitals for analysis. A lower ICU bed-to-intensivist ratio appears to improve septic shock outcomes. However, increasing the number of trained intensivists remains challenging due to the time, cost, and effort involved. The regionalization and centralization of intensivists should be considered to optimize efficiency and improve septic shock outcomes ^(29)^.

Our study population had several strengths in terms of selection compared to other studies. Most older adults in Japan are covered by social insurance. Therefore, disparities attributable to insurance plans were smaller than those reported in studies conducted in other countries. The small racial differences and the finely defined diagnostic codes for reimbursement in Japan help reduce bias. We identified patients with septic shock using a combination of diagnostic codes for sepsis and agents for infection and hypotension. In this study, the incidence of septic shock in older Japanese patients was 8.86 patients per 10,000 populations per year, which is almost equal to the rate reported in a recent epidemiologic study ^(2), (30)^.

However, our study has certain limitations due to the use of administrative data. First, we could not collect data on laboratory tests, vital signs, physical examination findings, or the stage of septic shock upon ICU admission. Consequently, we could not calculate severity scores such as the Sequential Organ Failure Assessment or Acute Physiology and Chronic Health Evaluation 2 (APACHE 2). In this study, the CCI was used as a substitute for measures that may reflect the severity and progression of sepsis. The CCI can predict mortality in ICU patients to a similar extent as a physiology-based score such as the Simplified Acute Physiology Score 2 (SAPS 2) ^(31)^.

Second, information on patients’ decisions regarding limitations of life-sustaining treatment was not available. We considered circumstances in which patients refused treatment just before or after ICU admission, or cases where physicians proposed withdrawing invasive treatment because they judged the patient was too ill to survive. By limiting inclusion to patients who stayed in the ICU for ≥2 days and were administered multiple agents (antibiotics and vasopressors), we assumed that all included patients were willing to receive treatment. Conversely, the intensity of therapeutic intervention may have been influenced by the use of ventilators and continuous renal replacement therapy, in addition to the selection of multidrug administration. This study did not include detailed treatment modalities as variables. Given that differences in treatment modalities may be associated with individual intensivists, they were not included as simultaneous explanatory variables in the analysis.

Third, we could not ascertain how physicians provided clinical services in the ICU. This assessment includes a combination of intensity, unit format, and the total number of ICU physicians and staff. No nationwide hospital surveys have reported the number of ICU physicians, intensivists, or closed units in Japan. Furthermore, the intensivists in our study may only be listed on the JSICM board-certified physician list and may not necessarily work in the ICU. Therefore, several factors must be considered, including the number of ICU physicians and the influence of intensivists. In Japan, staffing an ICU with a dedicated physician is difficult due to a shortage of physicians; thus, most ICUs are likely to have only 1 physician. As such, we assumed that the number of intensivists analyzed represented the maximum number of intensivists in the workforce at each hospital.

Fourth, the cause of death during the study period could not be determined. In our study population, death was likely due to aging or other comorbidities. We attempted to determine the timing of outcomes to ensure that extraneous factors were unlikely to influence mortality. Moreover, we considered the outcomes necessary to reflect the quality of care and evaluated the 28-day mortality rate after ICU admission.

Finally, given that this study was conducted in a specific region of Japan, there may be limitations in terms of external validity. Furthermore, the evidence regarding the presence of a regional context effect remains inconclusive. Fukuoka Prefecture contains a relatively large number of medical institutions and university hospitals. Conversely, hospital distribution across Japan varies significantly, with some regions having a single large hospital covering a wide geographical area. Consequently, a study employing data from a nationwide cohort with a larger sample size may better demonstrate regional context effects and exhibit greater generalizability. Nevertheless, we believe that variations in mortality rates among regions are possible, even within the modified scope of the target regions.

### Conclusions

Intensivists certified by the Society of Intensive Care Medicine appear to be more beneficial for older patients with septic shock in terms of 28-day mortality than physicians without such certification. Unlike ICU mortality in the case mix, septic shock mortality in older patients may be sensitive to the intensity of ICU physician involvement. Intensivists can contribute to improving septic shock mortality in older populations; therefore, a high- density of intensivists relative to ICU beds may be desirable. Moreover, continuous involvement in intensive care medicine may indirectly improve prognosis. Further investigation is required to confirm the robustness and accuracy of the relationship between physician background and patient outcomes.

## Article Information

### Conflicts of Interest

None

### Acknowledgement

The authors would like to thank the Wide-Area Association of Latter-Stage Elderly Healthcare of Fukuoka Prefecture for providing the healthcare claims database.

### Author Contributions

Conceptualization: Shinichiro Yoshida and Akira Babazono. Data curation: Shinichiro Yoshida. Formal analysis: Shinichiro Yoshida, Akira Babazono, and Ning Liu. Writing - original draft: Shinichiro Yoshida. Writing - review and editing: Shinichiro Yoshida and Akira Babazono. Supervision: Ning Liu, Reiko Yamao, and Reiko Ishihara. All authors interpreted the data, critically revised the manuscript for important intellectual content, and approved the final version of the manuscript.

### Approval by Institutional Review Board (IRB)

This study was approved by the Institutional Review Board of Kyushu University (Clinical Bioethics Committee of the Graduate School of Medical Sciences, Kyushu University [approval number 2021-335]), which waived the requirement for informed consent for this non-interventional study because information from an anonymized dataset was analyzed.

## Supplement

Supplementary Tables
