# Erratum: 2025;8(3): 708-17 (DOI: 10.31662/jmaj.2024-0331)

**DOI:** 10.31662/jmaj.e0003

**Published:** 2026-02-06

**Authors:** Shinichiro Yoshida, Akira Babazono, Ning Liu, Reiko Yamao, Reiko Ishihara

**Affiliations:** 1 Graduate School of Medical Sciences, Kyushu University, Fukuoka, Japan; 2 Department of Health Care Administration and Management, Faculty of Medical Sciences, Kyushu University, Fukuoka, Japan; 3 Department of Preventive Medicine and Community Health, University of Occupational and Environmental Health, Kitakyushu, Japan; 4 Department of Human Sciences, Faculty of Human Sciences, Osaka University of Economics, Osaka, Japan

Article: Yoshida S, Babazono A, Liu N, et al. Regional differences and mortality-associated risk factors among older patients with septic shock: administrative data analysis with multilevel logistic regression modelling. JMA J. 2025;8(3):708-17.

Correction 1: Table 4, Age group

Line 4 should be removed.

Correction 2: Table 4, CCI

Line 2 should be removed.

