# Supplementary material for: Regional Differences and Mortality-associated Risk Factors among Older Patients with Septic Shock: Administrative Data Analysis with Multilevel Logistic Regression Modelling
Source: JMA J. 2025 Jun 6;8(3):708–17. doi: 10.31662/jmaj.2024-0331 (PMC12329006; doi:10.31662/jmaj.2024-0331)
Supplement: Supplementary Tables [file 2433-3298-8-3-0708-s001.pdf]

Supplementary Table 1. Geographic profiles (2015)

SMA	Area, km²	Population, thousands	Population density/km²	No. of patients aged ≥75 years, thousands	Beneficiaries of healthcare insurance for late-stage older adults, thousands, n (%)	No. of ICUs	Gross regional (domestic) product, billion USD*
1	559	1635	2925	154	141 (91.8)	12	62.14
2	207	284	1372	27	25 (94.4)	1	10.99 **
3	173	155	899	20	19 (97.4)	1	
4	233	434	1858	41	40 (96.9)	3	10.12
5	468	456	975	59	57 (97.2)	5	11.72
6	562	133	237	21	21 (97.9)	1	3.84
7	369	181	491	28	26 (92.8)	1	4.26

8	601	1097	1824	157	150 (95.4)	10	33.54
9	569	184	324	27	26 (97.6)	2	5.88
10	366	84	229	13	13 (100.0)	0	3.33
11	264	223	846	40	38 (95.1)	0	5.78
12	252	109	434	18	17 (94.4)	0	3.82
13	364	126	347	22	19 (86.8)	0	2.45
Fukuoka Prefecture	4987	5101	1023	627	593 (94.6)	36***	157.86
Japan	377,972	127,110	341	16,126	15,597 (96.7)	661	4430.13

SMA secondary medical area

* The value was calculated as 1 USD = 120.13 JPY (the average rate in 2015).

** The sum of SMA 2 and SMA 3.

*** Includes one paediatric intensive care unit.

Supplementary Table 2. Disease names, codes, and corresponding ICD-10 codes used in this study

Disease name for health-insurance claims in Japan	Disease code for health insurance claims in Japan	Corresponding ICD-10 code
Salmonella sepsis	8834047	A021
Septicemic plague	8840047	A207
Anthrax sepsis	8837143	A227
Acute and fulminating melioidosis	8841155	A241
Erysipelothrix sepsis	8841141	A267
Extraintestinal yersiniosis	8830894	A282
Listerial sepsis	8840971	A327
Meningococcemia, unspecified	8835794	A394
Sepsis due to streptococcus, Group A	8845514	A400
Sepsis due to streptococcus, Group B	8849560	A401
Sepsis due to streptococcus, Group D	8846237	A402
Sepsis due to *Streptococcus pneumoniae*	8838800	A403
Sepsis due to streptococcus, Group C	8849563	A408
Sepsis due to streptococcus, Group G	8849566	

Sepsis due to haemolytic streptococcus	8840853	A409
Streptococcal sepsis	8841195	
Sepsis due to methicillin-resistant *Staphylococcus aureus*	8830124	A410
Sepsis due to *S. aureus*	8830966	
Sepsis due to coagulase-negative staphylococcus	8833325	A411
Sepsis due to methicillin-resistant coagulase-negative staphylococcus	8847071	
Staphylococcal sepsis	381001	A412
Sepsis due to *Haemophilus influenzae*	8830719	A413
Sepsis due to anaerobes	8833217	A414
Sepsis due to gram-negative bacillus	8832868	A415
Sepsis due to gram-negative organisms	8832870	
Sepsis due to gram-positive organisms	8847009	A418
Sepsis due to enterococcus	8847054	
Sepsis due to *Bacillus cereus*	8847109	
Sepsis	389004	A419

Sepsis due to in-hospital infection	389012	
Septic shock	7855015	
Septic pneumonia	8838823	
Actinomycotic sepsis	8840084	A427
Gonococcal sepsis	8841086	A548
Herpes viral sepsis	8839986	B007
Viral sepsis	8830759	B349
Candidal sepsis	8831569	B377
Progressive septic granulomatosis	8834976	D71
Septic pericarditis	8838821	I301
Septic endocarditis	8838820	I330
Septic pharyngitis	8838817	The020
Septic bronchitis	8838818	J209
Sepsis due to infection of tracheostomy	8832182	J950
Septic abscess	8838822	L029
Septic dermatitis	8838824	L080
Septic osteomyelitis	8838819	M869

Sepsis after abortion	8841001	O080
Septic shock after abortion	8841002	
Sepsis due to labour	8839900	O753
Puerperal sepsis	8834106	O85
Obstetric septic embolism	8834062	O883
Infection following a procedure	8835355	T814
Sepsis due to catheterization	8841319	
Infection following immunization	8840863	T880

ICD-10, *International Classification of Diseases, 10th revision* (World Health Organization, Geneva, Switzerland, 1994).

Supplementary Table 3. Results of multilevel logistic regression analysis on regional variations

**Variable**		**Univariate**		**Multivariate**	
		**OR (95% CI)**	***P***	**AOR (95% CI)**	***p***
Age group, y	75–79	Reference		Reference	
	80–84	1.20 (0.86–1.66)	0.29	1.28 (0.91–1.79)	0.16
	85–89	1.39 (0.99–1.94)	0.06	1.49 (1.05–2.11)	0.02*
	≥90	1.56 (1.05–2.32)	0.03*	1.86 (1.23–2.83)	<0.01*
Sex	Male	Reference		Reference	
	Female	0.77 (0.60–0.98)	0.04*	0.73 (0.56–0.94)	0.02*
Fiscal year	2015	Reference		Reference	
	2016	1.03 (0.70–1.52)	0.87	1.09 (0.73–1.63)	0.66
	2017	1.09 (0.74–1.60)	0.68	1.15 (0.77–1.71)	0.50
	2018	0.87 (0.59–1.28)	0.47	0.87 (0.59–1.29)	0.50
	2019	1.01 (0.69–1.50)	0.95	1.09 (0.73–1.62)	0.68
CCI	Mild	Reference		Reference	
	Moderate	1.07 (0.84–1.36)	0.61	1.09 (0.85–1.41)	0.49
	Severe	1.09 (0.33–3.59)	0.89	1.30 (0.38–4.41)	0.68

Procedure	Postoperative	0.63 (0.48–0.81)	<0.01*	0.62 (0.47–0.81)	<0.01*
Number of hospital beds	≥400	Reference		Reference	
	<400	1.08 (0.84–1.38)	0.57	0.82 (0.59–1.15)	0.26
Proportion of ICU beds to hospital beds	<1.5%	Reference		Reference	
	1.5–3%	1.16 (0.86–1.57)	0.32	1.22 (0.84–1.77)	0.29
	≥3%	0.92 (0.69–1.24)	0.59	0.93 (0.65–1.33)	0.69
ICU bed-to-board certified physician ratios	≤4	Reference		Reference	
	>4	1.18 (0.75–1.85)	0.47	1.41 (0.84–2.36)	0.20
	No certified physician	1.64 (1.06–2.53)	0.03*	2.25 (1.36–3.72)	<0.01*
Type of SICM fee	Resource rich	Reference		Reference	
	Standard	1.06 (0.81–1.37)	0.69	0.81 (0.57–1.16)	0.25

OR, odds ratio; CI, confidence interval; AOR, adjusted odds ratio; CCI, Charlson Comorbidity Index; ICU, intensive care unit; SICM, specialized intensive care management

* $p<0.05$.
